# Milling as a route to porous graphitic carbons from biomass

**DOI:** 10.1098/rsta.2020.0336

**Published:** 2021-11-01

**Authors:** R. D. Hunter, J. Davies, S. J. A. Hérou, A. Kulak, Z. Schnepp

**Affiliations:** ^1^ School of Chemistry, University of Birmingham, B152TT Birmingham, UK; ^2^ Department of Chemical Engineering, Imperial College, London SW72AZ, UK; ^3^ School of Chemistry, University of Leeds, Leeds LS2 9JT, UK

**Keywords:** biomass, carbon, sustainability

## Abstract

This paper reports a simple way to produce porous graphitic carbons from a wide range of lignocellulosic biomass sources, including nut shells, softwood sawdust, seed husks and bamboo. Biomass precursors are milled and sieved to produce fine powders and are then converted to porous graphitic carbons by iron-catalysed graphitization. Graphitizing the raw (unmilled) biomass creates carbons that are diverse in their porosity and adsorption properties. This is due to the inability of the iron catalyst precursor to penetrate the structure of dense biomass material. Milling enables much more efficient impregnation of the biomass and produces carbons with homogeneous properties. Lignocellulosic biomass (particularly waste biomass) is an attractive precursor to technologically important porous graphitic carbons as it is abundant and renewable. This simple method for preparing the biomass enables a wide range of biomass sources to be used to produce carbons with homogeneous properties.

This article is part of the theme issue ‘Bio-derived and bioinspired sustainable advanced materials for emerging technologies (part 2)’.

## 1. Introduction

Porous carbon materials have been widely explored for applications in energy devices such as batteries [1], supercapacitors [2] and fuel cells [3] due to their high surface areas and conductivity. Other research has focused on exploiting their tailorable surface chemistry for use in separation technologies, particularly as adsorbents for water treatment [4]. Given the large scale of many of these applications, it is necessary to be able to produce porous carbons with desired properties on a large scale and in a sustainable way. Biomass precursors are an attractive option for precursors to porous carbons as they are abundant and renewable. Furthermore, a large amount of biomass waste is produced every year from the agriculture and timber industries.

One option for producing porous carbons with attractive properties from biomass is a process called catalytic graphitization. This involves combining an organic precursor (often a biological material) with a metal source and pyrolysing it in an inert atmosphere. Various transition metals, including nickel, cobalt and iron, have been used to produce porous graphitic carbons [5], but iron is particularly attractive due to its high abundance and low toxicity. Aqueous iron salts such as iron nitrate and iron chloride have been used to produce graphitic carbon materials from a wide range of organic precursors, including small molecules such as glucose [6,7], synthetic [8] and natural polymeric species [9] and raw biomass such as sawdust [10–12]. During pyrolysis, the organic precursor decomposes to amorphous carbon and the iron precursor forms Fe or Fe_3_C nanoparticles which are believed to catalyse the conversion of amorphous carbon to graphitic carbon. The process is analogous to the chemical vapour deposition synthesis of carbon nanotubes except the catalyst produces graphitic nanostructures from solid amorphous carbon rather than a gaseous precursor such as acetylene [13].

Iron-catalysed graphitization has been used to produce a range of porous graphitic carbons and the nature of the resulting graphitic nanostructures varies between the systems. In some cases, the Fe/Fe_3_C nanoparticles become coated in onion-like layers of graphite [14]. Other precursors produce highly disordered nanotubes, where the Fe_3_C/Fe catalyst has been observed to ‘burrow’ through the amorphous carbon, leaving a graphitic nanotube behind [15]. The driving force for graphitization and the reason for such varied structures being produced is not yet understood although it is believed to be in part driven by structural and compositional differences in the precursors [7]. Lignocellulosic biomass takes many forms and compositions, depending on the plant species and the part of the plant, e.g. leaves versus stems. In addition to the organic components of lignin, cellulose and hemicellulose, many plants also contain inorganic components and in some cases the percentage mass of these can be significant (e.g. silica and other inorganic components make up 23.5% of the mass of rice husks) [16].

To develop scalable routes to technologically useful carbons, it is necessary to ensure that a method can be used consistently across a range of biomass sources with different structures and compositions. In the case of iron-catalysed graphitization, we have discovered that one of the main challenges is efficient dispersion of the iron catalyst throughout the initial organic precursor before pyrolysis. This can be challenging as many biomass sources can be very dense and/or hydrophobic. Effective methods of achieving maximum dispersion do exist and include hydrothermal pre-treatments [17] and templating methods [18]. However, in this paper we show that milling biomass to a fine powder can be used as a very simple method to produce carbons with consistent properties such as graphitic content and adsorption capacity. Milling of hard biomass precursors such as bamboo enables efficient mixing of the iron precursor with the biomass and significantly enhances the degree of graphitization, producing carbons with considerably higher porosity. The method produces carbons with similar properties from a wide range of biomass precursors such as nut shells, grasses, seed husks and woody biomass.

## 2. Results and discussion

**Figure 1.**
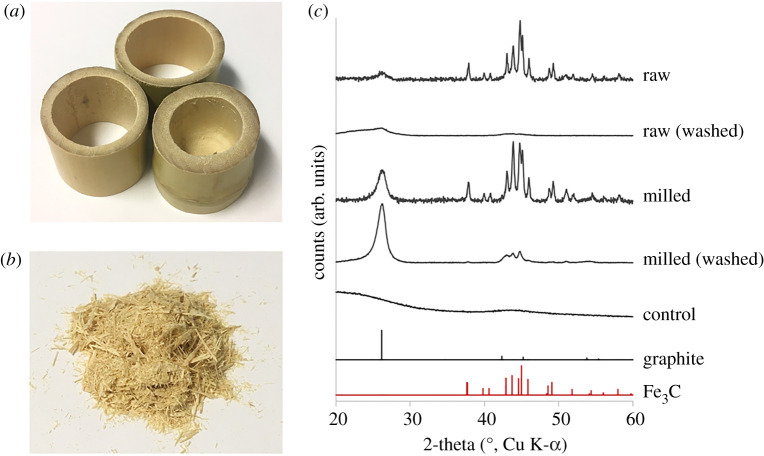
Images of (*a*) slices of bamboo stem approximately 5 cm in diameter and (*b*) milled and sieved bamboo. (*c*) PXRD patterns for carbons prepared by pyrolysing raw and milled bamboo that has been soaked in iron nitrate solution both before and after acid washing. (Online version in colour.)

As a first demonstration of the effectiveness of milling, porous carbons were prepared via iron-catalysed graphitization of bamboo. Slices of as-received bamboo stem (figure 1*a*) were roughly broken into pieces using a hammer: this sample was denoted ‘raw’ biomass. Some of the bamboo pieces were then milled to a fine powder and passed through a 150 µm sieve: this sample was denoted ‘milled’ biomass (figure 1*b*). To compare iron-catalysed graphitization fairly across both samples, it was necessary to keep a constant biomass : iron ratio. However, if the same volume of iron nitrate solution were added to both raw and milled bamboo, the raw bamboo would only absorb a small portion, with the remainder pooling in the bottom of the container. In order to ensure a homogeneous coating of iron nitrate across both samples, the maximum water absorption capacity for both the raw and milled biomass was determined. Raw bamboo absorbed 0.6 ml g^−1^ and milled bamboo was able to absorb 3.6 ml g^−1^, reflecting the higher surface area of the milled sample. Fresh samples of the bamboo were then combined with iron nitrate solution (0.4 g in 0.6 ml for raw bamboo and 0.4 g in 3.6 ml for milled bamboo). By varying the amount of water in this way, we ensured a homogeneous coating of the iron precursor over the whole sample while keeping the overall iron : biomass ratio constant. The samples were then dried and pyrolysed at 800°C to drive carbonization of the biomass, formation of the Fe_3_C catalyst nanoparticles and subsequent graphitization. Samples of both carbon products were then washed with dilute acid to remove the iron carbide catalyst.

Figure 1*c* shows powder X-ray diffraction (PXRD) patterns of the resulting carbons. The carbonized ‘raw’ bamboo shows clear evidence of formation of iron carbide (Fe_3_C, ICDD 00-035-0772). This results from decomposition of the iron nitrate precursor into iron oxide nanoparticles and subsequent reaction with the carbonized biomass. However, there is only a very small peak corresponding to the (002) reflection of graphitic carbon (ICDD 01-071-4630) and this is broad, indicating that only a small amount of disordered graphitic carbon has been produced. The XRD pattern for the acid-washed ‘raw’ bamboo again only shows a very low intensity peak for the (002) reflection of graphitic carbon that is overlaid with a broad hump at slightly lower 2*θ*. This is characteristic of turbostratic carbons and signifies a large degree of disorder within the carbon structure of this sample. The milled bamboo shows similar peaks for Fe_3_C but a much more intense peak for the (002) reflection, indicating that the Fe_3_C in the sample has catalysed the formation of graphitic nanostructures to a much greater extent. Acid washing removes most of the Fe_3_C but some peaks remain, indicating that some of the Fe_3_C nanoparticles are too deeply embedded for the acid to reach. This has been observed in our previous studies and is caused by the Fe_3_C nanoparticles being ‘trapped’ at the end of long graphitic nanotubes [12]. It should be noted that both the raw and milled biomass show slightly different peak profiles for Fe_3_C. This suggests the presence of some Fe, which is common as a byproduct of Fe_3_C formation and is believed to also act a graphitization catalyst. A control sample of bamboo that was pyrolysed without iron nitrate, shows only very broad humps consistent with an amorphous carbon structure. Further characterization of the porous carbons was carried out using Raman spectroscopy, which showed the presence of two prominent peaks at approximately 1325 and 1600 cm^−1^, corresponding to the D and G bands, respectively (electronic supplementary material, figure S2). The peak positions and broadening (electronic supplementary material, table S1) suggest nanocrystalline graphitic structures, indicating a high level of disorder in all of the samples. This is consistent with previous observations that iron-catalysed graphitic structures contain a lot of defects and are surrounded by regions of amorphous carbon [12].

**Figure 2.**
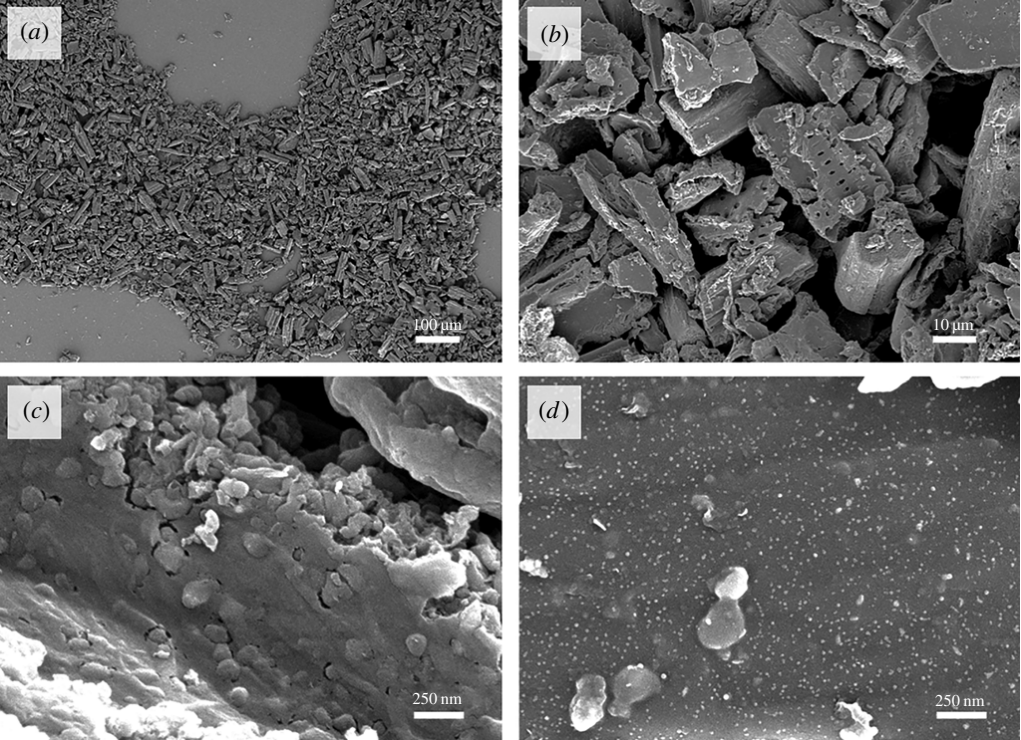
SEM images of (*a–c*) milled iron-graphitized bamboo at increasing magnification and (*d*) a control sample of bamboo that was pyrolysed without iron.

Scanning electron microscopy (SEM) of iron-graphitized milled biomass shows that the resulting carbon powder is composed of particles approximately 10–100 µm in length (figure 2*a*). Individual particles can be seen to have maintained small biological features (figure 2*b*). This is typical of carbonization of biomass, as biopolymers such as cellulose display high thermal stability and biomass samples undergo only limited shrinkage and deformation during pyrolysis. Higher magnification SEM images (figure 2*c*) show the graphitized bamboo contains rounded, bulbous structures, compared to the much smoother surface of the control sample. These features are typical of the graphitic shells and tubes that are commonly produced by iron-catalysed graphitization of biomass [12]. Importantly, these structures are not present in the control sample (figure 2*d*), showing that they are formed by the action of the iron catalyst.

**Figure 3.**
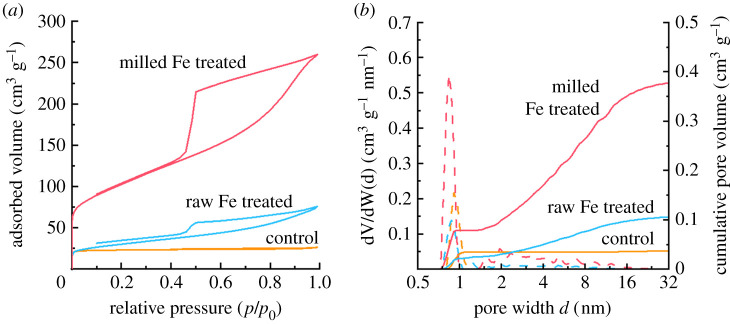
(*a*) Nitrogen sorption isotherms and (*b*) pore size distributions for acid-washed bamboo-derived carbons. Cumulative pore volumes are shown as full lines and differential pore volumes are displayed as dashed lines. (Online version in colour.)

Nitrogen sorption measurements for the control sample (bamboo pyrolysed without iron) shows a type I isotherm shape (figure 3*a*), suggesting a predominantly microporous material with a relatively low BET surface area of 92 m^2^ g^−1^. The sample displays a low pore volume of 0.04 cm^3^g^−1^, 99% of which arises from micropores (figure 3*b*). The lack of mesopores in this sample is expected due to the lack of any significant graphitic carbon features. The isotherm for the acid-washed iron-graphitized raw bamboo shows a type IV shape with hysteresis due to capillary condensation, consistent with the presence of mesopores. This sample has a similar BET surface area to the control sample (103 m^2^ g^−1^). However, the iron-graphitized bamboo shows a greater pore volume (0.11 cm^3^ g^−1^), with only 33% of the total volume being due to the presence of micropores, consistent with the hysteresis observed in the isotherm. These data (summarized in table 1) suggest that some mesopores are present in this sample and that a small amount of iron-catalysed graphitization has taken place, consistent with the PXRD data. Nitrogen sorption of the acid-washed iron-graphitized milled bamboo again shows a type IV isotherm, but there is a large increase in the BET surface area (356 m^2^ g^−1^) and total pore volume (0.39 cm^3^ g^−1^). The increase in adsorption at low *p*/*p*_0_ and the much larger hysteresis loop in the milled sample suggests an increase in porosity at both the micropore scale and the mesopore scale. This is consistent with the observation of a significant degree of iron-catalysed graphitization in this sample and indicates that milling the bamboo has maximized the ability of iron to produce graphitic mesopores through the sample. The closing of the hysteresis loops at approximately *p*/*p*_0_ = 0.4 suggests the presence of ink-bottle pores, which may indicate that the porous graphitic nanostructures are partially contracted or closed at the outer edges. Neither of the isotherms of the iron-graphitized bamboo carbons saturate at high *p*/*p*_0_, suggesting that the porosity may also extend past the measurable range of the instrument into the macropore range. It is worth reiterating at this point that the raw and milled bamboo was treated with the same iron : biomass ratio. The milling allows the iron precursor to coat the biomass more effectively, leading to more efficient iron-catalysed graphitization of the biomass, resulting in a more porous product.

**Table 1.** Adsorptive characteristics for bamboo-derived carbons, calculated from nitrogen sorption isotherms.

sample	*S*_BET_ (m^2^ g^−1^)	*S*_DR_ (m^2^ g^−1^)	*V*_tot_ DFT (cm^3^ g^−1^)	*V*_micro_/*V*_tot_ (%)
control (no Fe)	92	101	0.04	99
raw bamboo	103	116	0.11	32
milled bamboo	348	356	0.39	25

**Figure 4.**
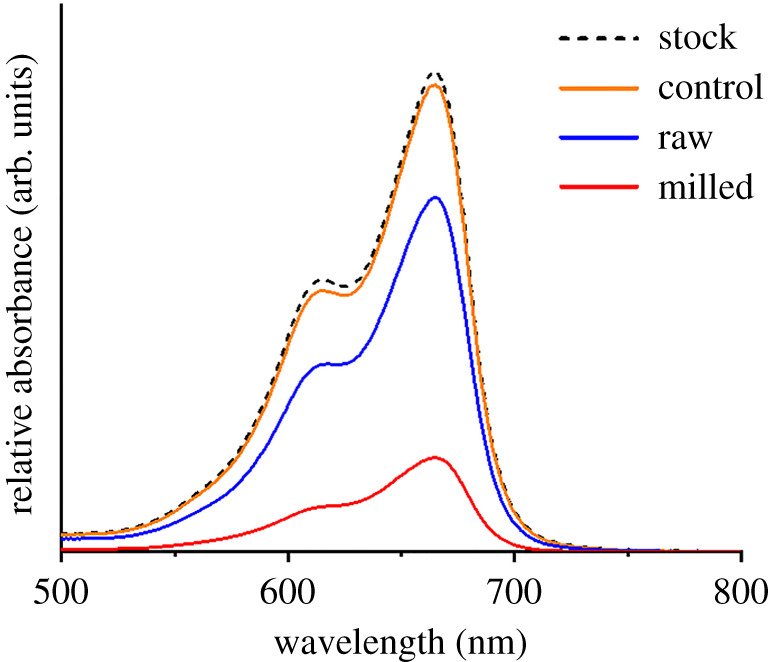
UV–Vis absorption spectra of methylene blue (stock) solution (2 × 10^−5^ m) after mixing with a control bamboo-derived carbon and acid-washed iron-graphitized raw and milled bamboo. (Online version in colour.)

To test the effect of the structural and textural changes achieved through milling of the bamboo precursor, the performance of the bamboo-derived carbons for the adsorption of methylene blue was examined. Methylene blue is commonly used to test adsorption capability of porous materials [19], and this is typically referred to as adsorptive capacity, measured in mg methylene blue adsorbed per g of adsorbent material. The control sample of raw bamboo that was pyrolysed in the absence of iron shows negligible absorption of methylene blue (figure 4), as would be expected from the low BET surface area determined by nitrogen sorption. Raw bamboo that was graphitized with iron (then acid washed) has a higher adsorption capacity for methylene blue (13 mg g^−1^). Again, this is expected from the increased BET surface area of this sample. The graphitized milled bamboo shows considerably higher adsorption capacity (59 mg g^−1^), a reflection of the high BET surface area for this sample and clear evidence that milling is an effective route to improving the porosity of the biomass-derived carbon.

**Figure 5.**
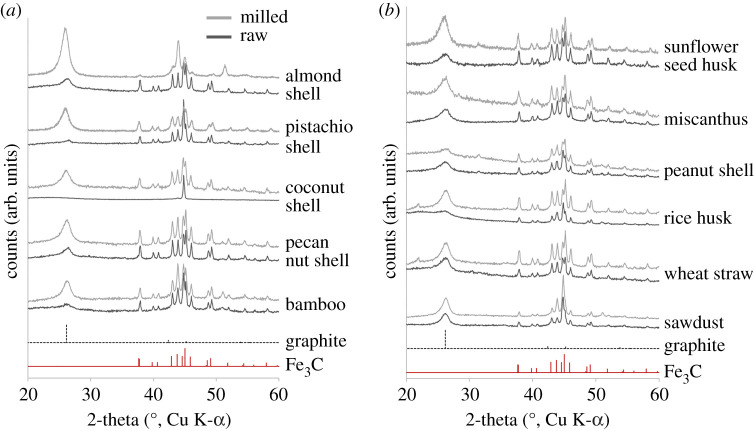
PXRD data for iron-graphitized carbons prepared from (*a*) ‘hard’ biomass where the absorption of water by the unmilled biomass was less than 1 ml g^−1^ and (*b*) ‘soft’ biomass where the absorption of water by the unmilled biomass was greater than 1 ml g^−1^. The upper pattern (light grey) for each sample shows the data for the milled sample, whereas the lower pattern (dark grey) shows data for the unmilled (raw) sample. (Online version in colour.)

**Figure 6.**
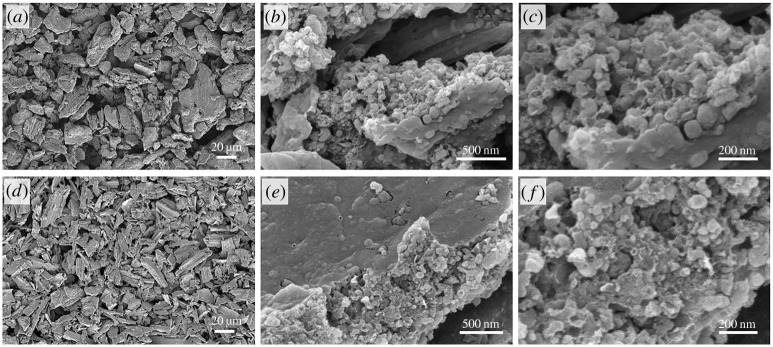
SEM images of (*a–c*) graphitized milled coconut shell and (*d–f*) graphitized milled miscanthus grass at various magnifications. Other biomass samples all showed similar microstructures.

To determine whether milling can be applied generally to produce porous graphitic carbons from biomass, we applied the same method to a range of other lignocellulosic biomass precursors. For each sample, the water absorption capacity was first determined for both raw and milled samples (table 2). One gram of raw or milled biomass was then saturated with a solution prepared with 0.4 g of iron nitrate in the required volume of water. This ensured that all samples received a homogeneous coating of the iron nitrate while maintaining a constant iron : biomass ratio for all samples. PXRD data for the resulting carbons (before acid washing) are shown in figure 5. The data are divided into two sections, including ‘hard’ biomass, where the precursor ‘unmilled’ biomass was very poor at absorbing water (less than 1 ml g^−1^) and ‘soft’ biomass, which already had good water absorption capability (greater than 1 ml g^−1^) before milling. The general trend is that the iron-graphitized carbons prepared from the ‘hard’ biomass samples show a more dramatic increase in the peak for the (002) reflection of graphitic carbon. Each of these samples absorbed a small amount of fluid (less than 1 ml g^−1^) and so the iron nitrate precursor was unable to penetrate into the sample. Milling the ‘hard’ biomass produced samples with a higher surface area, where the iron nitrate could be more finely dispersed through the biomass. The result was that a more significant amount of the sample could be graphitized. For most of the ‘soft’ biomass samples, there was still an increase in the amount of water that could be absorbed by the sample and subsequently an increase in intensity of the peak for the (002) reflection. However, the increase in graphitization was much less dramatic. The increase in the amount of fluid that could be absorbed was large in some cases, e.g. milled wheat straw could absorb over three times the amount of water as raw wheat straw. However, there was only a minor change in the degree of graphitization. This suggests that for samples that are highly absorbent, the benefit of milling is not so significant. One factor that we checked was the possibility that milling might be fractioning the samples. Since the milled samples were sieved, it was possible that larger ‘pieces’ that did not pass through the sieve may have contained higher levels of lignin. In order to check this, we also passed one sample through a 60 µm sieve. Coconut shell was chosen as this was one of the hardest biomasses we used in this study and had the greatest observable change between milled and non-milled. Electronic supplementary material, figure S1 shows the resulting XRD data and indicates that there is very little difference between 150 µm and 60 µm samples. This suggests that fractioning is minimal in this process. All milled graphitized samples showed similar microstructure in SEM images (figure 6), with rough surfaces and tube-like structures characteristic of catalytic graphitization.

**Table 2.** Volume of water absorbed by various biomass precursors (raw and milled).

	volume of water absorbed by the biomass precursor (ml g^−1^)	
type of biomass	raw	milled
almond shell granules	1	1.7
pistachio shells	0.4	3.4
coconut shell	0.8	1.9
pecan nut shell	0.7	3.3
bamboo	0.6	3.6
sunflower seed husks	2.4	4.2
miscanthus	2.5	4.5
peanut shells	3.2	6.5
rice husks	2.1	2.3
wheat straw	1.7	5.5
softwood sawdust	4.0	6.3

**Figure 7.**
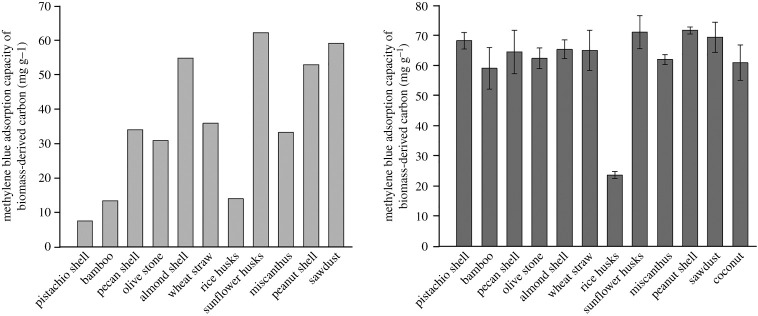
Adsorptive capacity for methylene blue for (*a*) raw and (*b*) milled acid-washed biomass-derived carbons.

To compare the porosity and adsorptive characteristics of all the graphitized raw and milled biomass samples, methylene blue adsorption studies were carried out (using acid-washed carbons). All graphitized biomass samples showed an increase in methylene blue adsorption when comparing the raw and milled samples. The adsorptive capacity varied greatly across the different graphitized raw biomass samples (7.6 mg g^−1^–62 mg g^−1^), as shown in figure 7*a*. For the graphitized milled biomass samples, there was considerably more consistency in methylene blue absorptive capacity, with values ranging from 59 mg g^−1^ to 72 mg g^−1^ (figure 7*b*). The only exception was rice husks, with a value of 24 mg g^−1^. There are multiple factors that will affect the porosity (and thus the adsorptive capacity) of the carbons produced from the different biomass samples. One of these may be the composition of the original biomass, including cellulose, hemicellulose, lignin and inorganic matter. Rice husks are known to contain high levels of silica [20] and so thermogravimetric analysis (in air to 600°C) was performed on a selection of the biomass samples in this study. The data showed that bamboo, sunflower seed husks and peanut shells had a small residual ‘ash’ content of 10%, 11% and 9%, respectively. By contrast, rice husks contained 33% residual ‘ash’. This high level of inorganic composition of the rice husks means that less carbon is available to be graphitized and may also inhibit graphitization by ‘blocking’ the path of the graphitization catalyst. The consistency in methylene blue absorptive capacity between all other milled graphitized biomass samples suggests that the cellulose/hemicellulose/lignin composition (which can vary quite substantially) has a minimal effect on graphitization. Electronic supplementary material, table S2 and figure S3 show the methylene blue adsorption capacity plotted against ash, cellulose and lignin content for a range of samples (compositions taken from literature) and no correlation is observable. The ability of the biomass precursor to be coated efficiently in the catalyst precursor, which is achieved readily by milling, is far more significant than any effect of biomass composition.

## 3. Conclusion

In this paper, we have shown that iron-catalysed graphitization can be applied to a variety of lignocellulosic biomass sources to produce porous graphitic carbon materials. Fe_3_C was present in all the iron-treated samples upon pyrolysis, suggesting that in all cases, this species was formed *in situ* and acted as the catalyst for converting the amorphous carbon intermediate into graphitic carbon. In the specific case of raw bamboo, it was shown that iron-catalysed graphitization increased the mesoporosity, BET surface and total pore volume of the carbon product compared to a control sample pyrolysed without iron. Milling the bamboo before graphitization produced a carbon with dramatically higher surface area and micro/mesoporosity. This is believed to be due to milling to a fine powder enabling enhanced impregnation of the biomass by the iron precursor solution. The adsorptive properties of graphitic carbons produced from a wide range of milled biomass samples were remarkably similar. This included both ‘soft’ biomass types such as grasses or sawdust and also hard/dense biomass such as nut shells. This demonstrates that milling can be applied as a simple method to achieve homogeneity in carbon products derived from a wide range of biomass sources.

## 4. Experimental set-up

### (a) Materials

Bamboo, coconut shells, miscanthus, peanut shells, pecan shells, pistachio shells, rice husks, wheat straw, almond shell granules, sunflower seed husks, olive stone granules and softwood sawdust were sourced from various suppliers and used ‘as received’ without extra drying. Iron (III) nitrate nonahydrate (CAS 7782-61-8) and hydrochloric acid (CAS 7647-01-0) were both sourced from Sigma-Aldrich. Raw biomass samples were used to produce carbons via the method below. A portion of each of the biomass samples was also milled using a Scheiben-Schwingmühle-TS heavy-duty disc mill. The resulting powder was then sieved through a 150 µm sieve to produce samples which have been denoted ‘milled’ throughout this paper.

### (b) Water absorption

In order to fairly compare the graphitization of different biomass samples, it was necessary to be able to keep a constant iron : biomass ratio. This was impossible if a standard iron nitrate stock solution was used as each biomass absorbed different amounts of solution. For poorly absorbing samples, the iron nitrate solution would just pool in the bottom of the container rather than coating the sample. Therefore, the water absorption capacity of each sample was determined by adding 0.1 ml aliquots and mixing for 5 min after each addition until a saturation point was reached (when pooling of water began to occur).

### (c) Catalytic graphitization

Fe(NO_3_)_3_.9H_2_O (0.4 g, 1 mmol) was dissolved in the required volume of DI water to achieve saturation of the biomass (table 2) and mixed with raw biomass (1 g). The mixtures were dried in an air oven at 70°C for 24 h and carbonized in a tube furnace at a heating rate of 5°C min^−1^ under a nitrogen atmosphere with a flow rate of 1 l min^−1^ to 800°C. The samples were held at 800°C for 1 h before cooling completely to room temperature.

### (d) Acid washing

In total, 0.2 g of carbon/Fe_3_C sample was added to 20 ml 0.1 M HCl solution and sonicated for 1 h. The mixture was then magnetically stirred for 24 h. The solid sample was collected by centrifugation (5 min, 10 000 r.p.m.) and washed three times with deionized water and once with ethanol, then left to dry at room temperature in air for 24 h. All adsorption studies were carried out on the acid-washed samples.

### (e) Powder X-ray diffraction

Samples were ground into a fine powder and placed on low background silicon wafer sample holders provided by PANalytical. PXRD experiments were performed using a PANalytical Empyrean diffractometer with a copper anode (wavelengths: *Kα*_1_ = 1.5406 Å, *Kα*_2_ = 1.5443 Å) and a Pixel 2D detector. The diffractometer did not have a monochromator but the K*_β_* radiation was removed with a nickel filter.

### (f) Scanning electron microscopy

Samples were mounted on an SEM stub using an adhesive copper tape. Samples were viewed with a FEG-SEM FEI Nova 450 using a CBS detector (detector of backscattered electrons), operating at 5 kV with deceleration mode.

### (g) Porosimetry

The N_2_ sorption analysis was performed at 77 K on a Micromeritics 3Flex instrument in the relative pressure range 10^−6^–0.99 *p*/*p*_0_ to avoid N_2_ condensation on the micrometer scale. The samples (50–70 mg) were degassed overnight at 200°C in a SmartVacPrep instrument and further degassed at 200°C for 300 min in the 3Flex instrument. The samples were analysed using 12 mm wide glass tubes and checkseals. The pore size distribution was obtained using the Microactive Software using the heterogeneous surface nonlinear DFT model (HS-NLDFT). The regularization factor was set at 10^−2^ to obtain a smooth curve, representative of the pore size of the material.

### (h) Methylene blue adsorption

In total, 0.01 g of carbon sample was added to 100 ml, 2 × 10^−5^ M methylene blue solution and magnetically stirred for 1 h. Solution was then filtered through a 45 µm syringe filter to remove the solid carbon. UV–Vis absorption spectroscopy was then performed using a Cary 50 UV–Vis spectrophotometer. The adsorptive capacity was calculated using the following equation:

4.1 $$q=\frac{V(C_{i}-C_{f})}{M}.$$

where *q* = adsorptive capacity (mg of methylene blue per g of carbon). *V* = volume of methylene blue solution (l); C_i_ = initial concentration of methylene blue solution (mg l^−1^); C_f_ = final concentration of methylene blue solution after adsorption (mg l^−1^); M = mass of carbon material (g).

## Data accessibility

All data are contained within the manuscript. Raw data e.g. for X-ray diffraction patterns will be available via the University of Birmingham data open access repository.

The data are provided in electronic supplementary material.
